# Identification of candidate genes and mutations in QTL regions for immune responses in chicken

**DOI:** 10.1111/age.12280

**Published:** 2015-03-05

**Authors:** M. Siwek, A. Slawinska, M. Rydzanicz, J. Wesoly, M. Fraszczak, T. Suchocki, J. Skiba, K. Skiba, J. Szyda

**Affiliations:** ^1^ Animal Biotechnology DepartmentUniversity of Technology and Life SciencesMazowiecka 2884‐085BydgoszczPoland; ^2^ Department of Human Molecular GeneticsInstitute of Molecular Biology and BiotechnologyAdam Mickiewicz UniversityUmultowska89 61‐614PoznańPoland; ^3^ Institute of GeneticsBiostatistics LabWroclaw University of Life SciencesKozuchowska 7Wroclaw51‐631Poland

**Keywords:** association, avian genome, immune trait, quantitative trait nucleotide, SNP

## Abstract

There are two categories of immune responses – innate and adaptive immunity – both having polygenic backgrounds and a significant environmental component. In our study, adaptive immunity was represented by the specific antibody response toward keyhole limpet hemocyanin (KLH); innate immunity was represented by natural antibodies toward lipopolysaccharide (LPS) and lipoteichoic acid (LTA). Defining genetic bases of immune responses leads from defining quantitative trait loci (QTL) toward a single mutation responsible for variation in the phenotypic trait. The goal of the reported study was to define candidate genes and mutations for the immune traits of interest in chicken by performing an association study of SNPs located in candidate genes defined in QTL regions. Candidate genes and SNPs in QTL regions were selected *in silico*. SNP association was based on a custom SNP panel, GoldenGate genotyping assay (Illumina) and two statistical models: random mixed model and CAR score. The most significant SNP for immune response toward KLH was located in the *JMJD6* gene located on GGA18. Four SNPs in candidate genes *FOXJ1* (GGA18), *EPHB1* (GGA9), *PTGER4* (GGAZ) and *PRKCB* (GGA14) showed association with natural antibodies for LPS. A single SNP in *ITGB4* (GGA18) was associated with natural antibodies for LTA. All associated SNPs mentioned above showed additive effects.

## Introduction

### Genetic bases of immune responses

Immune responses fall into the category of complex or quantitative traits and, as such, they are controlled by multiple genes with different magnitudes of phenotypic effects, along with the impact of the environment. Genomic regions related to the complex traits are defined as quantitative trait loci (QTL). Deciphering genetic bases of complex traits lead from defining of the QTL toward pointing at a single mutation responsible for a considerable amount of the genetic trait variation called a QTN (quantitative trait nucleotide). Molecular dissection from QTL to QTN demands several steps: QTL validation in independent populations, QTL fine mapping, *in silico* selection of positional and biological candidate genes, selection of SNP (single nucleotide polymorphism) markers located within candidate genes, and finally, an association study of SNPs with phenotypes of interest possibly resulting in QTN identification. Availability of genomewide SNP panels accelerated identification of causal mutations associated with economically important traits in livestock (Dekkers 2012).

### Immune responses

Immune response is composed of innate and adaptive responses. In our analysis, innate immunity was represented by natural antibodies (NAbs). Natural antibodies are immunoglobulins that need no exogenous stimulation of the immune system to be secreted by B‐1 cells in large quantities (Ochsenbein *et al*. 1999). NAbs are very effective as a first barrier to pathogen invasion, mostly due to their massive presence in the host organism and polyreactivity (Frank 2002). Therefore, NAbs are considered to be a crucial immune barrier at the initial steps of the immune response, before the acquired antibodies are generated (Siwek & Knol 2005). NAbs bind different highly conserved, homologous epitopes (homotopes), for example, lipopolysaccharides (LPS), the molecule found in the outer membrane of Gram‐negative bacteria, or lipoteichoic acid (LTA), which is an ingredient of cell walls of Gram‐positive bacteria. In our studies, adaptive immunity was represented by specific antibody response toward keyhole limpet hemocyanin (KLH). KLH is a copper‐containing, high‐molecular‐weight protein antigen collected from the hemolymph of the sea mollusk, *Megathura crenulata*. KLH is commonly used as a soluble model protein known to induce a Th‐2‐like response (Bliss *et al*. 1996). KLH is never encountered by birds during their lifetime; therefore, it represents a novel antigen, suitable for measuring primary immune responses.

### QTL regions

A QTL for a primary antibody response toward KLH (Siwek *et al*. 2003) and the QTL for NAbs for LPS and LTA (Siwek *et al*. 2006) were first detected in an experimental chicken population created by crossing two chicken lines divergently selected for a primary antibody response toward sheep red blood cells (Bovenhuis *et al*. 2002). Linkage analysis harbored four chicken chromosomes: GGA9 (QTL for Nabs/LPS), GGA14 (QTL for KLH and NAbs/LTA), GGA18 (QTL for NAbs/LPS) and GGAZ (QTL for NAbs/LPS). Subsequently, all these QTL were validated in two independent experimental populations: a cross of two chicken lines expressing different feather pecking behavior (Siwek *et al*. 2003, 2006) and in a cross of White leghorn and Green‐legged Partridgelike (Siwek *et al*. 2010; Slawińska *et al*. 2011). Validated QTL on the GGA9, GGA14 and GGA18 chromosomes were fine mapped using two statistical tools: meta QTL analysis and joined QTL analysis (Slawinska & Siwek 2013). The additional statistical analysis allowed for narrowing down of the QTL confidence intervals. These selected regions in the genome (on GGA9, GGA14, GGA18 and GGAZ) are hypothesized to contain causative mutations underlying genetic variation of innate and adaptive immune responses. Therefore, the goal of the reported study was to define candidate genes and mutations for the immune responses in chickens by performing an association study of the SNPs located in candidate genes.

## Methods

### Animals and phenotypic data

Analysis was carried out in an experimental population, created by crossing two breeds of hens: Green‐legged Partridgelike and White Leghorn. Animals were kept on a floor system on a farm at the University of Life Sciences in Lublin, Poland. All chickens were vaccinated according to the routine vaccination schedule, which incorporated a vaccine against Salmonella, Gumboro disease, bronchitis, Bourse Fabricius disease and encephalomyelitis. Population details are given in Siwek *et al*. (2010). The final F_2_ generation consisted of 506 individuals obtained in six hatches. Immune responses were defined as specific antibody response to KLH (SAb‐KLH) and natural antibodies (NAb) to the environmental antigens LPS and LTA. Phenotypic data were expressed by titers as the log_2_ values of the highest dilution giving a positive reaction as described by Siwek *et al*. (2003) (for KLH) and by Siwek *et al*. (2006) (for LTA and LPS). Descriptive statistics of the phenotypic data are given in Table 1.

**Table 1:** Descriptive statistics of keyhole limpet hemocyanin‐specific antibody titers (KLH SPAb), lipopolysaccharide (LPS) and lipoteichoic acid (LTA) natural antibody (NAb) titers in the blood serum of an F_2_ generation of WL × GP chicken crossbreds (*n *= 491)

Trait	Mean^a^	Min^b^	Max^c^
KLH	10.93 (2.89)	2.8	16.7
LPS	2.9 (1.30)	1	8.8
LTA	5.6 (1.75)	1	12

a Mean: mean value of KLH SPAb titers and LPS and LTA NAb titers. Standard deviations are given in parentheses.

b Min: minimum value of KLH SPAb titers and LPS and LTA NAb titers.

c Max: maximum value of KLH SPAb titers and LPS and LTA NAb titers.

### *In silico* gene/SNP selection

*In silico* analysis of positional and functional candidate genes covered four QTL regions associated with anti‐LPS, anti‐LTA natural antibodies and anti‐KLH specific antibodies and located on four chromosomes: GGA9, GGA14, GGA18 and GGAZ. The functions of the genes were subsequently determined based on NCBI, KEGG and Gene Ontology databases. Based on the Biomart (Ensembl) and Genecards (Stelzer *et al*. 2011) databases, SNPs were selected in coding regions of candidate genes. SNP selection was based on chicken genome build 3.1 (March 2012). Selected SNPs were subsequently analyzed according to Illumina's technical note for the Custom Golden Gate Genotyping Assay.

Selected regions in the chicken genome contained a total of 617 genes and 2023 SNPs. The analysis of the gene function reduced the initial number to 36 genes related to the innate and adaptive immune system, located on the following chromosomes: eight genes on GGA9 (*EPHB1, PROCR, KLHL6*,* GPC1, SOX14, ST6GAL1, PARL, ADIPOQ*), 14 genes on GGA14 (*CARD11, MAP2K3, TNFRSF13B, IL9R, IL21R, IL20RB, NLRC3, SOCS1, MAPK8IP3, PRKCB, PGP, TRAF7, PDGFA, SMURF1*) and seven genes each on GGA18 (*MAP2K4, JMJD6, SPHK1, FOXJ1, CRLF3, ITGB4, UNC13D*) and GGAZ (*PTGER4, JAK2, FGF10, IL31RA, IL6ST, PIK3R1, AP3B1*). The initial list of SNPs located in positional candidate genes was reduced to 384 due to the size limitations of the custom assay SNP panel.

### DNA isolation and SNP analysis

Genomic DNA, which was used as a matrix for the genotyping of SNP markers, was isolated from blood cells. DNA isolation was performed using a commercial kit, MasterPure DNA Purification Kit for Blood (Epicentre^®^). The DNA extraction procedure recommended by the manufacturer was modified due to the presence of nucleated erythrocytes in the chicken blood.

An Illumina custom 384‐plex oligonucleotide pool assay was designed, and the GoldenGate^™^ Genotyping Assay (Illumina Inc.) was conducted on 50 ng of genomic DNA according to manufacturer's protocol. Interplate replicates were included as quality control measurements of the overall genotyping experiment. Error rate was below 0.1%. Genotypes were assigned and annotated using genomestudio (Illumina Inc.) with a default SNP call threshold of 0.25. Samples with a call rate below 0.90 were reproduced or excluded from the study. SNPs with gen train scores <0.4 were zeroed.

### SNP effect estimation

The following random mixed model (RMM) was used to estimate the additive effects of the SNPs:

(1) y=Xμ+Zq+e,

where *y* represents a value of a considered trait; *X* is a design vector consisting of 1s; *μ* is a general mean; *Z* is a design matrix for SNP genotypes, which is parameterized as −1, 0 or 1 for a homozygous, heterozygous and an alternative homozygous SNP genotype respectively; *q* is a vector of random additive SNP effects; and *e* is a vector of residuals with $e∼N(0,I{\hat{\sigma }}_{e}^{2})$, where *I* is an identity matrix. The covariance structure of *q* was assumed to be $q∼N\left(\frac{0,I{\hat{\sigma }}_{e}^{2}}{N_{\mathrm{snp}}}\right)$, with *I* being an identity matrix, ${\hat{\sigma }}_{a}^{2}$ representing the additive genetic variance of a given trait estimated by a linear mixed model with a random animal polygenic effect and N_SNP_ being the number of SNPs used (here, 211).

The estimation of parameters of the above model was based on solving the mixed model equation (Henderson 1984):

(2) $$\left[\begin{array}{c} \hat{\mu }\\ \hat{q} \end{array}\right]={\left[\begin{array}{c} X^{T}R^{-1}XX^{T}R^{-1}Z\\ Z^{T}R^{-1}XZ^{T}R^{-1}Z+G^{-1} \end{array}\right]}^{-1}\left[\begin{array}{c} X^{T}R^{-1}y\\ Z^{T}R^{-1}y \end{array}\right],$$

with *R* represented by $I{\hat{\sigma }}_{e}^{2}$ and *G* represented by $\frac{I{\hat{\sigma }}_{a}^{2}}{N_{\mathrm{snp}}}$. The iteration on data technique was based on the Gauss–Seidel algorithm with residuals update (Legarra & Misztal 2008). Consequently, the variance of *y* is then given by *ZGA* ^*T*^ *+ R*.

For testing the significance (*H*_0_: *q*_i_ = 0 vs. *H*_1_: *q*_i_ ≠ 0) of the *i*th SNP effects in *q*, the Wald test was used. The test statistic follows under *H*_0_ a normal distribution with mean 0 and variance 1. Test statistics have the following form: $W=\frac{{\hat{q}}_{i}}{SE\left({\hat{q}}_{i}\right)}$, where $\text{SE}({\hat{q}}_{i})$ is a standard error of the *i*th estimated SNP effect $\hat{q_{i}}$.

### SNP association based on CAR score

The CAR score, a highly effective criterion for variable ranking in linear regression based on Mahalanobis decorrelation of the explanatory variables, proposed by Zuber & Strimmer (2011), was selected as one method to identify a subset of significant SNPs. According to Zuber & Strimmer (2011), this approach is very effective computationally. The CAR scores, *ω*i, defined as ω = P^−1/2^P_*Xy*_ were considered the SNP selection criterion, where P denotes the empirical correlation matrix among SNPs, and P_*Xy*_ is the marginal correlation vector between phenotype data and SNPs. Generally, CAR scores can be interpreted as something between marginal correlations and a standardized regression coefficient. In this model, the null distribution of the empirical CAR scores, used for obtaining type I error rates for SNPs, was defined as $Beta\left({\hat{\omega }}_{i}^{2},\frac{s}{2},\frac{s\left(N-2\right)}{2}\right)$, where *N* is the number of SNPs and $s=\sum_{i}{\hat{\omega }}_{i}^{2}$. The CAR criterion was evaluated using the r package CARE (R Core Team 2012). The disadvantage of this method seems to be the possibility of overemphasis of a set of SNPs which are close to this significance. This might be because different genes will have different number of SNPs, and moreover, the correlation between SNPs that are close to one another is high. If such a situation occurs, it is a rather marginal problem.

### Selection of the most significant SNP

SNPs selected as significant by the above two models were subjected to testing in order to select polymorphisms with possible QTN effects. A mixed model: y = *μ* + X_1_ snp + X_2_ sex +  X_2_ hatch + Zg + e, where *μ* is the overall mean, snp is a vector of fixed additive effects of SNPs representing polymorphisms selected as significant by the two previously applied methods, sex is a vector of fixed effects of sex, and hatch is a vector of fixed effects of six hatches; *X*_*i*_ represents corresponding design matrices, *g* is a random additive polygenic effect which follows $q∼N(0,A{\hat{\sigma }}_{a}^{2})$ with an additive polygenic covariance matrix between individuals *A* and the corresponding design matrix *Z,* and *e* represents a residual. Statistically, testing for QTN effect of the *i*th SNP corresponds to testing the following hypotheses H_0_: snp_i_ = 0 against H_1_: snp_i_ ≠ 0, which was performed by comparing the likelihood of the full model defined above (L_f_) with the likelihood of a reduced model with one SNP removed (L_r_) using the likelihood ratio test: *λ* = −2( ln *L*_*r*_ − ln *L*_*f*_) with an asymptotic distribution following $\chi_{1df}^{2}$. Testing was carried out separately for each trait. Nominal *P*‐values were subjected to Bonferroni's correction for multiple testing within traits. Furthermore, to test for dominance effects of the SNPs which showed significant additive effects, a likelihood of the above mixed model with additive effect of the SNPs and a likelihood of the above mixed model with additive and dominance effects were compared separately for each significant SNP, using *λ*. Moreover, for traits for which more than one SNP was identified as significant, significance of an effect of pairwise additive‐by‐additive epistasis was tested. The effects of QTN models were estimated using proc mixed in sas software (SAS Institute 2002–2004).

### Pathway analysis

The relation between immune responses to three antigens and the associated most significant candidate genes was analyzed using biograph (VIB Genetic Service Facility, University of Antwerp; Liekens *et al*. 2011). Based on this tool, and gene function defined in GeneCards (2013), several functional relations between our traits of interest and their candidate genes were proposed.

## Results

### Dataset

Each individual was genotyped using the custom assay SNP panel, which consists of 384 SNP markers. Forty SNP markers were removed from the set due to genotyping failure with the Golden Gate genotyping assay. In the final analysis, the SNP selection criteria were applied based on minor allele frequency (MAF), with a cutoff of 0.01, and genotyping quality, with a minimum call rate of 95%. After quality control, 211 SNPs were used in the final analysis, 132 SNPs were removed based on MAF criterion and one SNP was removed based on low call rate. The average MAF was 0.17 for all genotyped SNPs and 0.27 for SNPs selected for further analysis. The average call rate obtained for our dataset was high and amounted to 97.52% for all SNPs and 97.80% for selected SNPs.

### Candidate genes

The results of the candidate gene association analysis under the RMM and CAR score are presented in Table 2. Altogether, seven candidate genes for an antibody response to KLH, located on two chicken chromosomes, were selected with the mixed model: five genes located on GGA14 (*CARD11, IL9R, MAPK8IP3, PDGFA, PRKCB*) and two genes on GGA18 (*ITGB4, UNC13D*). Association with LPS response was shown for two genes on GGA9 (*EPHB1, PROCR*), two genes on GGA18 (*CRFL3, FOXJ1*) and one gene on GGAZ (*PTGER4*). Association with LTA response was shown for eight genes altogether: four genes on GGA14 (*IL9R, MAPK8IP3, PRKCB, MAP2K3*), three genes on GGA18 (*FOXJ1, ITGB4, JMJD6*) and one gene on GGAZ (*PTGER4*). The CAR score indicated a higher number of candidate genes associated with the traits of interest than did the mixed model, showing associations of an additional 16 genes: seven genes associated with immune response to KLH [one on GGA14 (*TRAF7*) and three each on GGA9 (*EPHB1, KLHL6, PROCR*) and GGA18 (*FOXJ1, JMJD6, MAP2K4*)]; five candidate genes associated with innate immune response to LPS [one gene on GGA9 (*KLHL6*) and four genes on GGA14 (*IL9R, MAPK8IP3, PRKCB, SOCS1*)]; and four genes associated with innate immune response to LTA [two genes on GGA14 (*CARD11, TNFRSF13B*) and one gene each on GGA9 (*KLHL6*) and GGA18 (*ITGB4*)].

**Table 2:** Association study of the candidate genes located on four chicken chromosomes (GGA9, GGA14, GGA18, GGAZ) and three immune traits [immune response to keyhole limpet hemocyanin (KLH), natural antibodies for lipopolysaccharide (LPS) and lipoteichoic acid (LTA)], based on two statistical approaches: a random mixed model (RMM) showing additive effects of significant SNPs and a nonparametric CAR score (CAR)

Trait	Gene ID	Gene name	Chromosome	Model	
				RMM	CAR
KLH	*EPHB1*	*EPH receptor B1*	9	NS	+
	*KLHL6*	*Kelch‐like 6 (Drosophila)*	9	NS	+
	*PROCR*	*Protein C receptor, endothelial*	9	NS	+
	*CARD11*	*Caspase recruitment domain family, member 11*	14	0.1028 0.0821	+
	*IL9R*	*Interleukin 9 receptor*	14	0.1025	+
	*MAPK8IP3*	*Mitogen‐activated protein kinase 8 interacting protein 3*	14	0.0932 0.1315	+
	*PDGFA*	*Platelet‐derived growth factor alpha polypeptide*	14	0.0868	+
	*PRKCB*	*Protein kinase C, beta*	14	0.0708	+
	*TRAF7*	*TNF receptor‐associated factor 7, E3 ubiquitin protein ligase*	14	NS	+
	*FOXJ1*	*Forkhead box J1*	18	NS	+
	*JMJD6*	*Jumonji domain containing 6*	18	NS	+
	*MAP2K4*	*Mitogen‐activated protein kinase kinase 4*	18	NS	+
	*ITGB4*	*Integrin, beta 4*	18	0.1197 0.0902	+
	*UNC13D*	*Unc‐13 homolog D (C. elegans)*	18	0.0841	+
LPS	*EPHB1*	*EPH receptor B1*	9	0.0313 0.0380 0.0373	+
	*PROCR*	*Protein C receptor, endothelial*	9	0.0439	+
	*KLHL6*	*Kelch‐like 6 (Drosophila)*	9	NS	+
	*IL9R*	*Interleukin 9 receptor*	14	NS	+
	*MAPK8IP3*	*Mitogen‐activated protein kinase 8 interacting protein 3*	14	NS	+
	*PRKCB*	*Protein kinase C, beta*	14	NS	+
	*SOCS1*	*Suppressor of cytokine signaling 1*	14	NS	+
	*CRLF3*	*Cytokine receptor‐like factor 3*	18	0.0366	+
	*FOXJ1*	Forkhead box J1	18	0.0378	+
	*PTGER4*	*Prostaglandin E receptor 4 (subtype EP4)*	Z	0.0630	+
LTA	*KLHL6*	*Kelch‐like 6 (Drosophila)*	9	NS	+
	*IL9R*	*Interleukin 9 receptor*	14	0.0591	+
	*MAPK8IP3*	*Mitogen‐activated protein kinase 8 interacting protein 3*	14	0.0444 0.0597	+
	*PRKCB*	*Protein kinase C, beta*	14	0.0467	+
	*MAP2K3*	*Mitogen‐activated protein kinase kinase 3*	14	0.0493	+
	*CARD11*	*Caspase recruitment domain family, member 11*	14	NS	+
	*TNFRSF13B*	*Tumor necrosis factor receptor superfamily, member 13B*	14	NS	+
	*FOXJ1*	Forkhead box J1	18	0.0413	+
	*ITGB4*	*Integrin, beta 4*	18	0.0595	+
	*JMJD6*	*Jumonji domain containing 6*	18	0.0414	+
	*ITGB4*	*Integrin, beta 4*	18	NS	+
	*PTGER4*	*Prostaglandin E receptor 4 (subtype EP4)*	Z	0.0484	+

+, gene selected with CAR score model.

### Selection of most significant SNPs within candidate genes

Polymorphisms with the most significant effects, most likely representing QTNs, are summarized in Table 3. No dominance or epistasis was detected. For immune response to LPS, an additive effect of four SNPs within four candidate genes located on different chromosomes [GGA9 (*EPHB1*), GGA14 (*PRKCB*), GGA18 (*FOXJ1*) and GGAZ (*PTGER4*)] was identified. For immune response to KLH and LTA, there were two single SNPs (*JMJD6* and *ITGB4*) with additive effects, both located on GGA18.

**Table 3:** The most significant SNPs in candidate genes located on four chicken chromosomes (GGA9, GGA14, GGA18, GGAZ) and three immune traits [immune response to keyhole limpet hemocyanin (KLH), natural antibodies for lipopolysaccharide (LPS) and lipoteichoic acid (LTA)]

Trait	SNP ID	Genome build	Chromosome	Gene ID	Gene name
KLH	rs15820324	3.1	18	*JMJD6*	*Jumonji domain containing 6*
LPS	rs14110239	3.1	18	*FOXJ1*	*Forkhead box J1*
	rs15946185	3.1	9	*EPHB1*	*EPH receptor B1*
	rs16102750	3.1	Z	*PTGER4*	*Prostaglandin E receptor 4 (subtype EP4)*
	rs15731101	3.1	14	*PRKCB*	*Protein kinase C, beta*
LTA	rs14110519	3.1	18	*ITGB4*	*Integrin, beta 4*

## Discussion

Our original linkage analysis harbored five QTL located on four chicken chromosomes: QTL for immune response toward LPS on GGA9, GGA18 and GGAZ and QTL for immune response toward LTA and KLH, both located on GGA14 (Siwek *et al*. 2010; Slawińska *et al*. 2011). The current study showed that the selected SNPs were located within regions originally suggested by those linkage analyses. But, surprisingly, it also pointed to other genomic regions. Candidate genes for KLH association are located on GGA9 (*EPHB1, KLHL6, PROCR*) and GGA18 (*ITGB4, UNC13D, MAP2K4, FOXJ1, JMJD6*). Association with immune response toward LPS was directed toward genes located on GGA14 *(MAPK8IP3, IL9R, SOCS1*,* PRKCB*). Candidate genes for LTA association are located on GGA9 (*KLHL6*), GGA18 (*FOXJ1, ITGB4, JMJD6*) and GGAZ (*PTGER4*). There are three possible explanations for this phenomenon. The linkage analysis was based on a limited number of microsatellite markers; therefore, the power of that analysis was much lower than for the association study. Selected candidate genes might play a role in both types of immune responses: innate and adaptive. The linkage analysis in the current population was a validation study, oriented toward particular QTL regions detected in other experiments.

### Differences between models

In the first step of the analysis, significant SNPs were pre‐selected using two different approaches of an underlying inheritance mode: a mixed model, assuming additive effects of SNPs, and the CAR score, a model‐free approach without any assumptions on the genetic effect of the SNP. Moreover, in the mixed model, a (normal) distribution is superimposed on the estimates, and consequently, it resulted in a lower number of significant SNPs. On the other hand, the CAR score applies no shrinkage.

### Function of candidate genes

Associated genes are involved in regulation of B‐cell and T‐cell proliferation (*CARD11*), B‐cell activation (*PRKCB*), cytokine–cytokine receptor interaction (*IL9R*,* CRLF3*), MAPK signaling pathway (*MAPK8IP3*,* PDGFA*,* EPHB1*,* MAP2K3*,* MAP2K4*), defense response to virus (*UNC13D*), antigen processing and presentation (*PROCR*), humoral immune response, negative regulation of B‐cell activation (*FOXJ1*), activation of T‐cell factor signaling (*PTGER4*), cell surface receptor signaling pathway, macrophage activation, T‐cell differentiation in thymus (*JMJD6*), B‐cell receptor signaling pathway (*KLHL6*), cytokine‐inducible negative regulators of cytokine signaling (*SOCS1*), humoral immunity by interacting with a TNF ligand (*TNFRSF13B*), encoding the integrin beta 4 subunit (a receptor for the laminins) (*ITGB4*) and signal transduction for members of the TNF receptor superfamily (*TRAF7*).

Zakharova *et al*. (2009) indicated that *JMJD6* (*jumonji domain containing 6* gene) serves as a membrane‐associated receptor that regulates phagocytosis in immature macrophages and is expressed in the cytosol and nucleus of mature macrophage‐like cells. This metabolic activity is closely related to KLH's mode of action. It has been shown that KLH induces Th2 immune response and production of IL‐4, IL‐5, IL‐ 10 and IL‐13 cytokines, which promote alternative macrophage activation (Bliss *et al*. 1996; Allen & Wynn 2011).

LPS activates innate immune response by interaction with its specific toll‐like receptor 4 (TLR4). TLR4 triggers a Th1 type of adaptive immune response, which activates macrophages and participates in the generation of Tc cells, resulting in a cell‐mediated immune response. Products of three of the four most significant candidate genes associated with LPS immune responses (*FOXJ1, EPHB1, PTGER4*) are involved in T‐cell regulation or proliferation:

- *FOXJ1* (*forkhead box J1*) participates in the regulation of T‐cell tolerance, inhibition of the spontaneous autoimmunity and regulating thymic egress (Srivatsan & Peng 2005).
- *EPHB1* (*EPH receptor B1*) encodes Eph kinases, which are the largest family of receptor tyrosine kinases. Eph kinases and their ligands (ephrins) are expressed on the surface of T cells, B cells and monocytes/macrophages (Yu *et al*. 2004). A role of EPHB1 in immune response has been documented experimentally by Luo *et al*. (2011), who observed reduced thymus and spleen size and cellularity in double null mutated *Ephb1* and *Ephb2* mice as well as a significant decrease in the double‐positive and single‐positive thymocyte subpopulations and mature CD4 and CD8 cells in the periphery in double knockout mice.
- *PTGER4* (*prostaglandin E receptor 4*) can dramatically modulate immune response given that prostaglandin E2 production is enhanced during inflammation. Generally, cellular immune response regulation is under the control of distinct EP receptors from which EP4 regulates antigen presenting cell functions (Nataraj *et al*. 2001).

Moreover, *protein kinase C, beta* (*PRKCB*), another candidate gene indicated as being related to LPS immune response in our study, is involved in B‐cell survival and antigenic response. B cells respond to TLR ligands and present antigen (Lund 2008), organize the structure of lymphoid tissues and regulate lymphangiogenesis. An experiment on mice deficient in protein kinase C beta demonstrated an essential role of this gene in BCR‐induced glycolysis in B cells (Blair *et al*. 2012).

*ITBG4* is the most significant gene associated with LTA immune responses in our study. LTA initiates immune response through a very particular pattern recognition receptor: toll‐like receptor 2 (TLR2). TLRs are known to interact with macrophages or dendritic cells, known also as antigen presentation cells (APC). Airway epithelial cells have been demonstrated to be accessory APCs, capable of activating T cells, whereas silencing of *ITGB4* resulted in impaired antigen presentation and suppressed T‐cell proliferation (Liu *et al*. 2012).

### Pathways analysis

**Figure 1:**
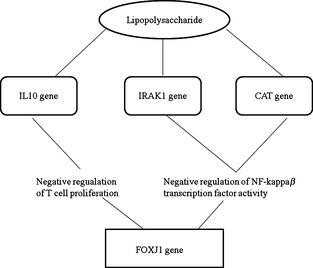
Pathway analysis between lipopolysaccharide (LPS) and the *FOXJ1* gene (adopted from Biograph.be).

**Figure 2:**
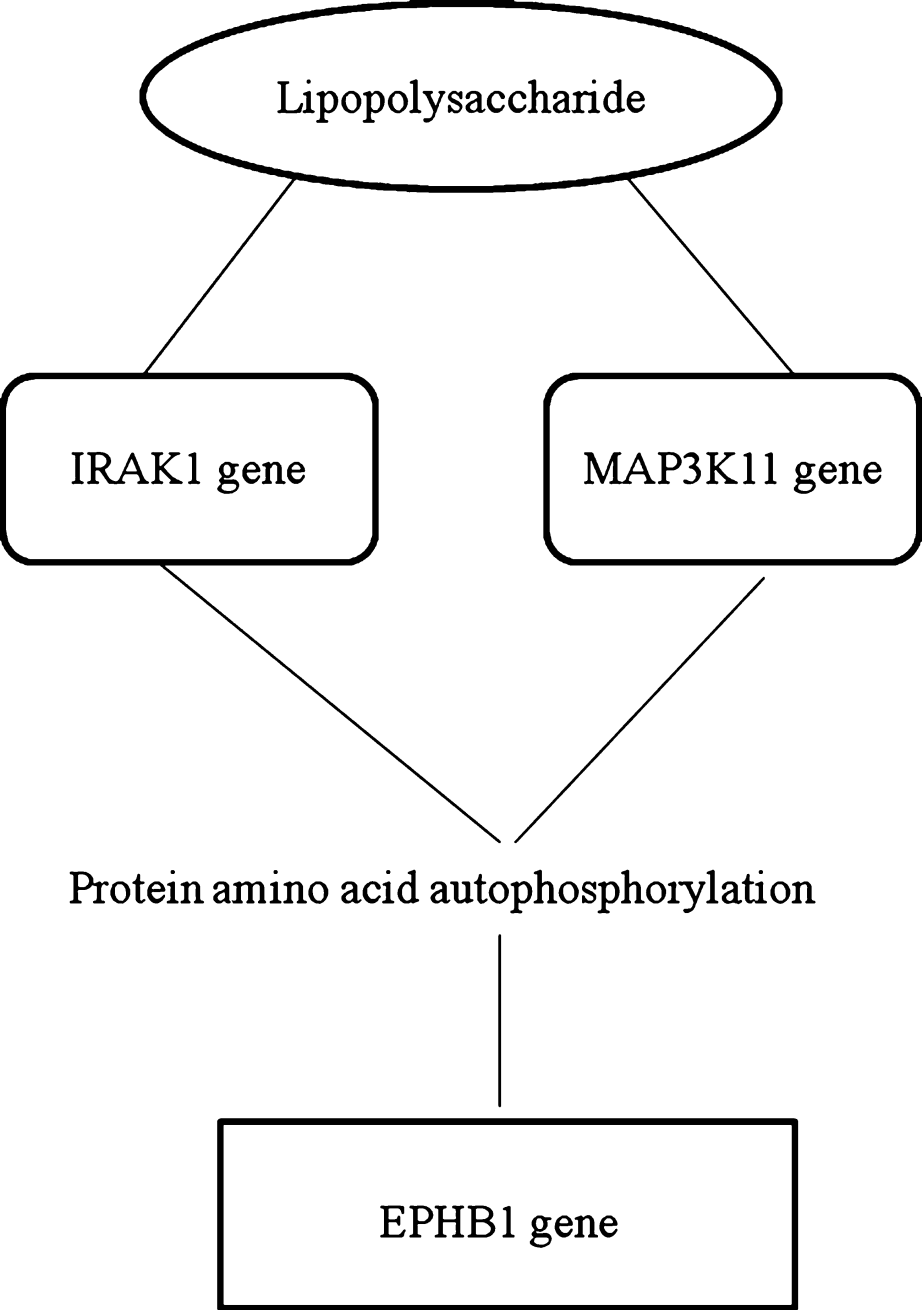
Pathway analysis between lipopolysaccharide (LPS) and the *EPHB1* gene (adopted from Biograph.be).

Results indicate that a relation between an immune response to LPS of bacterial origin and the *FOXJ1* gene can go through different pathways such as LPS – *IL10* gene – negative regulation of T‐cell proliferation – *FOXJ1*, or LPS – *IRAK1* gene/*CAT* gene – negative regulation of NF kappa*β* transcription factor activity – *FOXJ1* (Fig. 1). The relation between immune response to LPS and the *PTGER4* gene is direct and goes through inflammation. Another connection between LPS and the *EPHB1* gene goes through *IRAK1/MAP3K11* – protein amino acid autophosphorylation – *EPHB1* (Fig. 2). The *IRAK1* gene (*interleukin‐1 receptor‐associated kinase 1*) encodes interleukin‐1 receptor‐associated kinase 1, one of the two putative serine/threonine kinases that become associated with the interleukin‐1 receptor (IL1R) upon stimulation. Serine/threonine protein kinase plays a critical role in initiating innate immune response against foreign pathogens. These kinases are involved in TLR and IL‐1R signaling pathways. The *CAT* gene (*catalase*) encodes a protein which promotes growth of cells, including T cells and B cells.

**Figure 3:**
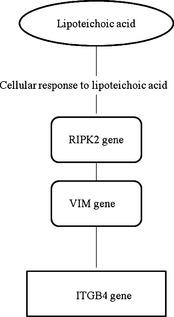
Pathway analysis between lipoteichoic acid (LTA) and the *ITGB4* gene (adopted from Biograph.be).

The immune response relation between LTA and *ITGB4* leads through the *RIPK2* gene and *VIM* gene. Protein encoded by the *RIPK2 (receptor‐interacting serine‐threonine kinase 2*) gene contains a C‐terminal caspase activation and recruitment domain and is a component of signaling complexes in both the innate and adaptive immune pathways. *VIM* (vimentin) encodes a protein which is involved in the immune response (Fig. 3).

**Figure 4:**
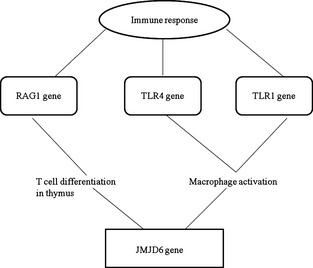
Pathway analysis of immune responses and the *JMJD6* gene (adopted from Biograph.be).

There is no direct information proposed by BioGraph on the relation between KLH and the *JMJD6* gene. However, the link between immune response and the *JMJD6* gene goes through various pathways: *RAG1* – T‐cell differentiation in thymus or TLR4/TLR1 – macrophage activation. *RAG1* (*recombination activating 1* gene) encodes a protein involved in the activation of immunoglobulin V‐D‐J recombination (Fig. 4).

## Conclusions

Revealing a genetic architecture of immune responses toward KLH, LTA and LPS led to three general conclusions. First, the most significant SNPs for immune responses toward KLH and LTA are located outside the QTL regions originally proposed by linkage analysis, which indicates its relatively poor resolution. Also, in the search for causal mutations in candidate genes, a linkage analysis can be well regarded as a preliminary tool but not as an indicator of final results. Second, innate and adaptive immunity have some genes in common. Third, immunity predominantly follows an additive mode of inheritance.

## References

[age12280-bib-0001] Allen J.E. & Wynn T.A. (2011) Evolution of Th2 immunity: a rapid repair response to tissue destructive pathogens. PLoS Pathogens 7, e1002003. 21589896 10.1371/journal.ppat.1002003 PMC3093361

[age12280-bib-0002] Blair D. , Dufort F.J. & Chiles T.C. (2012) Protein kinase C*β* is critical for the metabolic switch to glycolysis following B‐cell antigen receptor engagement. Biochemical Journal 15, 165–69. 22994860 10.1042/BJ20121225

[age12280-bib-0003] Bliss J. , Van Cleave V. , Murray K. , Wiencis A. , Ketchum M. , Maylor R. , Haire T. , Resmini C. , Abbas A.K. & Wolf S.F. (1996) IL‐12, as an adjuvant, promotes a T helper 1 cell, but does not suppress a T helper 2 cell recall response. Journal of Immunology 156, 864–87. 8558014

[age12280-bib-0004] Bovenhuis H. , Bralten H. , Nieuwland M.G.B. & Parmentier H.K. (2002) Genetic parameters for antibody response of chickens to sheep red blood cells based on selection experiment. Poultry Science 81, 309–15. 10.1093/ps/81.3.309 11905446

[age12280-bib-0005] Dekkers J.C. (2012) Application of genomics tools to animal breeding. Current Genomics 13, 207–12. 23115522 10.2174/138920212800543057 PMC3382275

[age12280-bib-0006] Ensembl/BioMart : http://www.ensembl.org/biomart/martview/4c9e9118b2e0e70021327ab55e698083

[age12280-bib-0007] Frank S.A. (2002) Specificity and cross‐reactivity In: Immunology and Evolution of Infectious Diseases (Ed. by Frank S.A.), pp. 33–54. Princeton University Press, 41 William Street, Princeton, New Jersey, 08540, United Kingdom. 20821852

[age12280-bib-0008] Henderson C.R. (1984) Applications of Linear Models in Animal Breeding, 3rd ed (Ed. by Schaeffer L.R.) University of Guelph, Guelph, ON.

[age12280-bib-0009] Legarra A. & Misztal I. (2008) Technical note: computing strategies in genome‐wide selection. Journal of Dairy Science 91, 360–6. 18096959 10.3168/jds.2007-0403

[age12280-bib-0010] Liekens A.M.L. , De Knijf J. , Daelemans W. , Goethals B. , De Rijk P. & Del‐Favero J. (2011) biograph: unsupervised biomedical knowledge discovery via automated hypothesis generation. Genome Biology 12, R57. 21696594 10.1186/gb-2011-12-6-r57 PMC3218845

[age12280-bib-0011] Liu C. , Qin X. , Liu H. & Xiang Y. (2012) Downregulation of integrin *β*4 decreases the ability of airway epithelial cells to present antigens. PLoS One 7, e32060. 22545078 10.1371/journal.pone.0032060 PMC3335869

[age12280-bib-0012] Lund F.E. (2008) Cytokine‐producing B lymphocytes – key regulators of immunity. Current Opinion Immunology 20, 332–8. 10.1016/j.coi.2008.03.003 PMC2474694 18417336

[age12280-bib-0013] Luo H. , Charpentier T. , Wang X. , Qi S. , Han B. , Wu T. , Terra R. , Lamarre A. & Wu J. (2011) Efnb1 and Efnb2 proteins regulate thymocyte development, peripheral T cell differentiation, and antiviral immune responses and are essential for interleukin 6 (IL‐6) signaling. Journal of Biology and Chemistry 286, 41135–52. 10.1074/jbc.M111.302596 PMC3308828 21976681

[age12280-bib-0014] Nataraj C. , Thomas D.W. , Tilley S.L. , Nguyen M.T. , Mannon R. , Koller B.H. & Coffman T.M. (2001) Receptors for prostaglandin E(2) that regulate cellular immune responses in the mouse. Journal Clinical Investigations 108, 1229–35. 10.1172/JCI13640 PMC209534 11602631

[age12280-bib-0015] Ochsenbein A.F. , Fehr T. , Lutz C. , Suter M. , Brombacher F. , Hengartner H. & Zinkernagel R.M. (1999) Control of early viral and bacterial distribution and disease by natural antibodies. Science 286, 2156–9. 10591647 10.1126/science.286.5447.2156

[age12280-bib-0016] R Core Team . r: A Language and Environment for Statistical Computing. R Foundation for Statistical Computing, Vienna, Austria ISBN 3‐900051‐07‐0, URL 2012, http://www.R-project.org/

[age12280-bib-0017] SAS Institute Inc. (2002‐2004) sas 9.1.3, SAS Institute Inc, Cary, NC.

[age12280-bib-0018] Siwek M. & Knol E.F. (2005) Genetic aspects of biological processes underlying the defense system in the neonate. Folia Biologica, 53(Supplement 1), 39–43.16212106

[age12280-bib-0019] Siwek M. , Buitenhuis A.J. , Cornelissen S.J.B. , Nieuwland M.G..B. , Bovenhuis H. , Crooijmans R.P.M.A. , Groenen M.A.M. , De Vries‐Reilingh G. , Parmentier H.K. & Van der Poel J.J. (2003) Detection of different QTL for antibody response to keyhole lympet hemocynain and *Mycobacterium butyricum* in two unrelated populations of laying hens. Poultry Science 82, 1842–5. 10.1093/ps/82.12.1845 14717541

[age12280-bib-0020] Siwek M. , Buitenhuis B. , Cornelissen S. , Nieuwland M. , Knol E.F. , Crooijmans R. , Groenen M. , Parmentier H. & van der Poel J. (2006) Detection of QTL for innate: non‐specific antibody levels binding LPS and LTA in two independent populations of laying hens. Developmental Comparative Immunology 30, 659–66. 16368139 10.1016/j.dci.2005.09.004

[age12280-bib-0021] Siwek M. , Slawinska A. , Nieuwland M. , Witkowski A. , Zięba G. , Minozzi G. , Knol E.F. & Bednarczyk M. (2010) A quantitative trait locus for a primary antibody response to keyhole limpet hemocyanin on chicken chromosome 14—Confirmation and candidate gene approach. Poultry Science 89, 1850–7. 10.3382/ps.2010-00755 20709969

[age12280-bib-0022] Slawinska A. & Siwek M. (2013) Meta – and combined – QTL analysis of different experiments on immune traits in chickens. Journal of Applied Genetics 54, 483–7. 24114202 10.1007/s13353-013-0177-6 PMC3825546

[age12280-bib-0023] Slawińska A. , Witkowski A. , Nieuwland M. , Minozzi G. , Bednarczyk M. & Siwek M. (2011) Quantitative trait loci associated with the humoral innate immune response in chickens were confirmed in a cross between Green‐Legged Partridgelike and White Leghorn. Poultry Science 90, 1909–15. 10.3382/ps.2011-01465 21844254

[age12280-bib-0024] Srivatsan S. & Peng S.L. (2005) Cutting edge: Foxj1 protects against autoimmunity and inhibits thymocyte egress. Journal of Immunology 175, 7805–9. 10.4049/jimmunol.175.12.7805 16339515

[age12280-bib-0025] Stelzer G. , Dalah I. , Iny Stein T. *et al* (2011) *In‐silico* human genomics with GeneCards. Human Genomics, 5, 709–17 22155609 10.1186/1479-7364-5-6-709 PMC3525253

[age12280-bib-0026] Yu G. , Luo H. , Wu Y. & Wu J. (2004) EphrinB1 is essential in T‐cell–T‐cell co‐operation during T‐cell activation. Journal of Biology Chemistry 279, 55531–9. 10.1074/jbc.M410814200 15502157

[age12280-bib-0027] Zakharova L. , Dadsetan S. & Fomina A.F. (2009) Endogenous *Jmjd6* gene product is expressed at the cell surface and regulates phagocytosis in immature monocyte‐like activated THP‐1 cells. Journal of Cell Physiology 221, 84–91. 10.1002/jcp.21829 19492415

[age12280-bib-0028] Zuber V. & Strimmer K. (2011) High‐dimensional regression and variable selection using CAR scores. Statistical Applications in Genetics and Molecular Biology 10, 34.

